# Supplementary material for: Evaluation of Self-Assembly Pathways to Control Crystallization-Driven Self-Assembly of a Semicrystalline P(VDF-co-HFP)-b-PEG-b-P(VDF-co-HFP) Triblock Copolymer
Source: Molecules. 2020 Sep 3;25(17):4033. doi: 10.3390/molecules25174033 (PMC7504740; doi:10.3390/molecules25174033)
Supplement: Supplementary file 1 [file molecules-25-04033-s001.pdf]

SUPPLEMENTARY MATERIAL

Evaluation of Self-Assembly Pathways to Control Crystallization-Driven Self-Assembly of a Semicrystalline P(VDF-*co*-HFP)-*b*-PEG-*b*-P(VDF-*co*-HFP) Triblock Copolymer

Enrique Folgado[1,2], Matthias Mayor[2], Vincent Ladmiral[1*] and Mona Semsarilar[2*]

[1] ICGM, Univ Montpellier, CNRS, ENSCM, Montpellier, France.

[2] IEM, Univ Montpellier, CNRS, ENSCM, Montpellier, France.

* Correspondence: vincent.ladmiral@enscm.fr; mona.semsarilar@umontpellier.fr

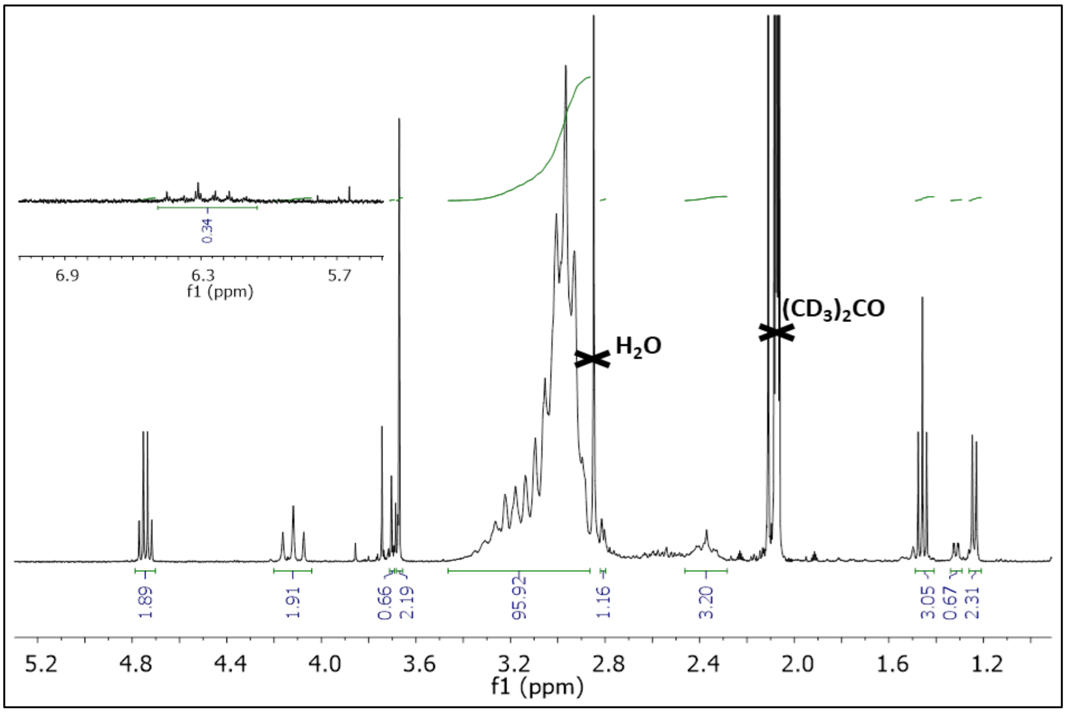

Figure S1. ^{1}H NMR spectrum ($(CD_3)_2CO$, 300 MHz) of P(VDF_{51}-*co*-HFP_4)-XA

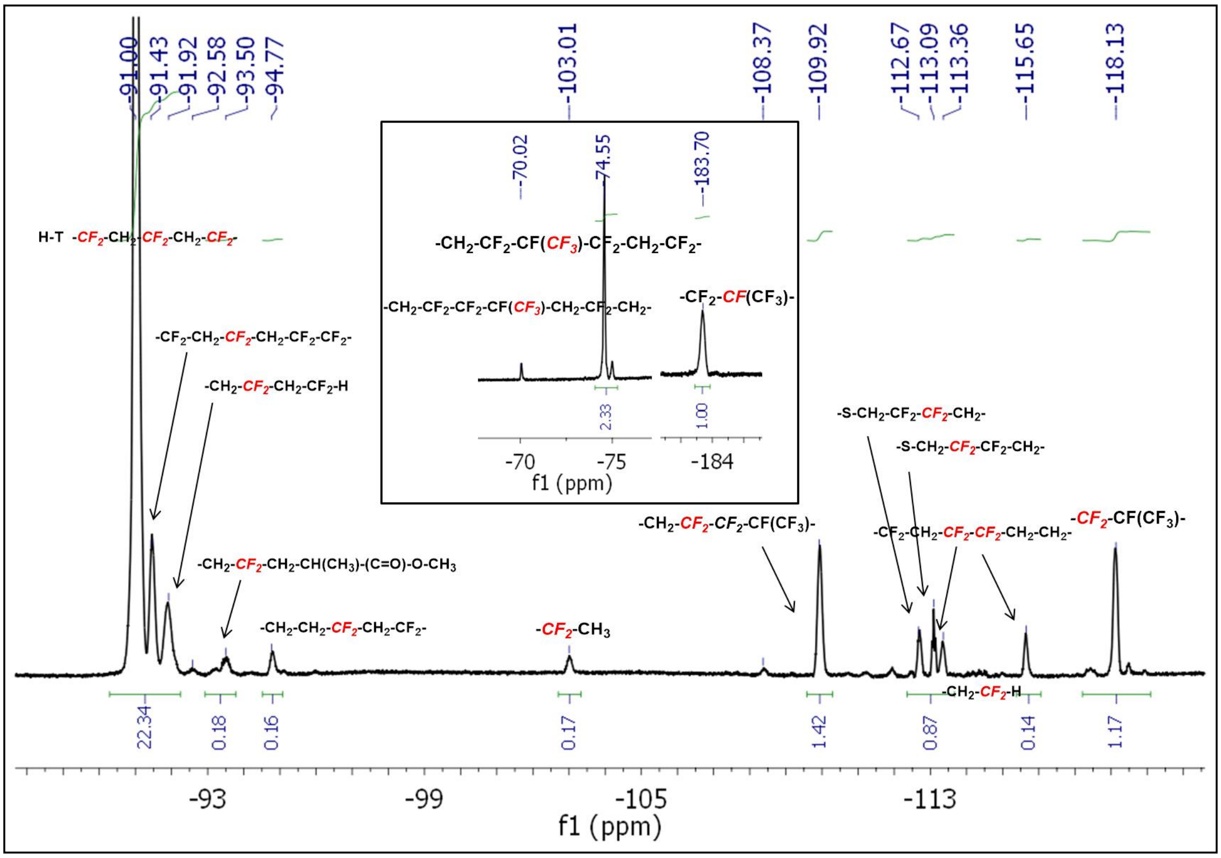

Figure S2. ^{19}F NMR spectrum ($(CD_3)_2CO$, 282 MHz) of P(VDF$_{51}$-*co*-HFP$_4$)-XA

S3. VDF and HFP %mol determination from ^{19}F NMR

*(values extracted from Fig. S2)

$$\%\,mol\,VDF = \frac{\sum \int CF_2/_2}{\sum \int CF_2/_2 + \int CF} \times 100 \tag{1}$$

With:

$$\sum \int CF_2 = \int_{-90.3}^{-91.7} CF_2\,(HT) + \int_{-91.7}^{-92.3} CF_2H + \int_{-92.9}^{-93.8} CF_2\,(R\ end\ group) + \int_{-94.6}^{-95.0} CF_2\,(HT) + \int_{-112.4}^{-113.7} CF_2(Z\ end\ group + HH) + \int_{-115.5}^{-115.9} CF_2\,(HH) \tag{2}$$

$$\% \, mol \, VDF = \frac{\frac{22.34 + 0.18 + 0.16 + 1.42 + 0.87 + 0.14}{2}}{\frac{22.34 + 0.18 + 0.16 + 1.42 + 0.87 + 0.14}{2} + 1.00} = \mathbf{92.6}$$

(3)

$$\% \, mol \, HFP = 100 - 92.6 = \mathbf{7.4}$$

(4)

S4. DP of VDF and DP of HFP determination from ^{1}H NMR data.

*(values extracted from Figure S1)

$$DP_{VDF} = \frac{\int_{2.70}^{3.19} \mathrm{CH_2\ (HT)} + \int_{2.28}^{2.43} \mathrm{CH_2(TT)} \int_{4.02}^{4.17} + \mathrm{CH_2(End\ Group)}}{\frac{2}{3} \times \int_{1.19}^{1.24} \mathrm{CH_3\ (R - CTA)}} =$$

$$DP_{VDF} = \frac{95.92 + 3.20 + 1.91}{\frac{2}{3} \times 3} = \mathbf{50.5}$$

$$DP_{HFP} = \frac{DP_{VDF} \times \%mol_{HFP}}{\%mol_{VDF}} = \mathbf{4.0}$$

S5. P(VDF-*co*-HFP) M_n Determination from NMR data

$$M_{n\,NMR} = M_n CTA + (DP_{VDF} \times M_n \, VDF) + DP_{HFP} \times M_n \, HFP$$

$$M_{nNMR} = 208.3 + 50.5 \times 64.03 + 4.0 \times 150.02 = \mathbf{4041.90 \; g/mol}$$

With M_nCTA = 208.3 g/mol, M_n VDF = 64.03 g/mol and, M_n HFP = 150.02 g/mol.

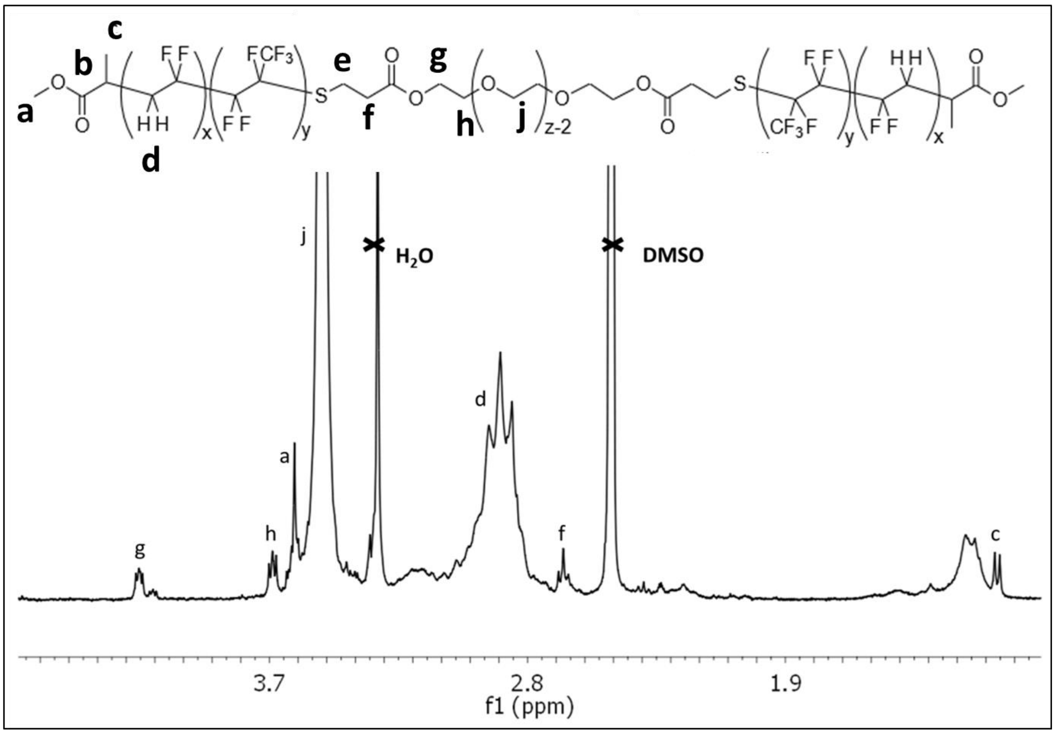

Figure S6. ^{1}H NMR spectrum ($(CD_3)_2SO$, 400 MHz) of P(VDF$_{51}$-*co*-HFP$_4$)-*b*-PEG$_{136}$-*b*- P(VDF$_{51}$-*co*-HFP$_4$).

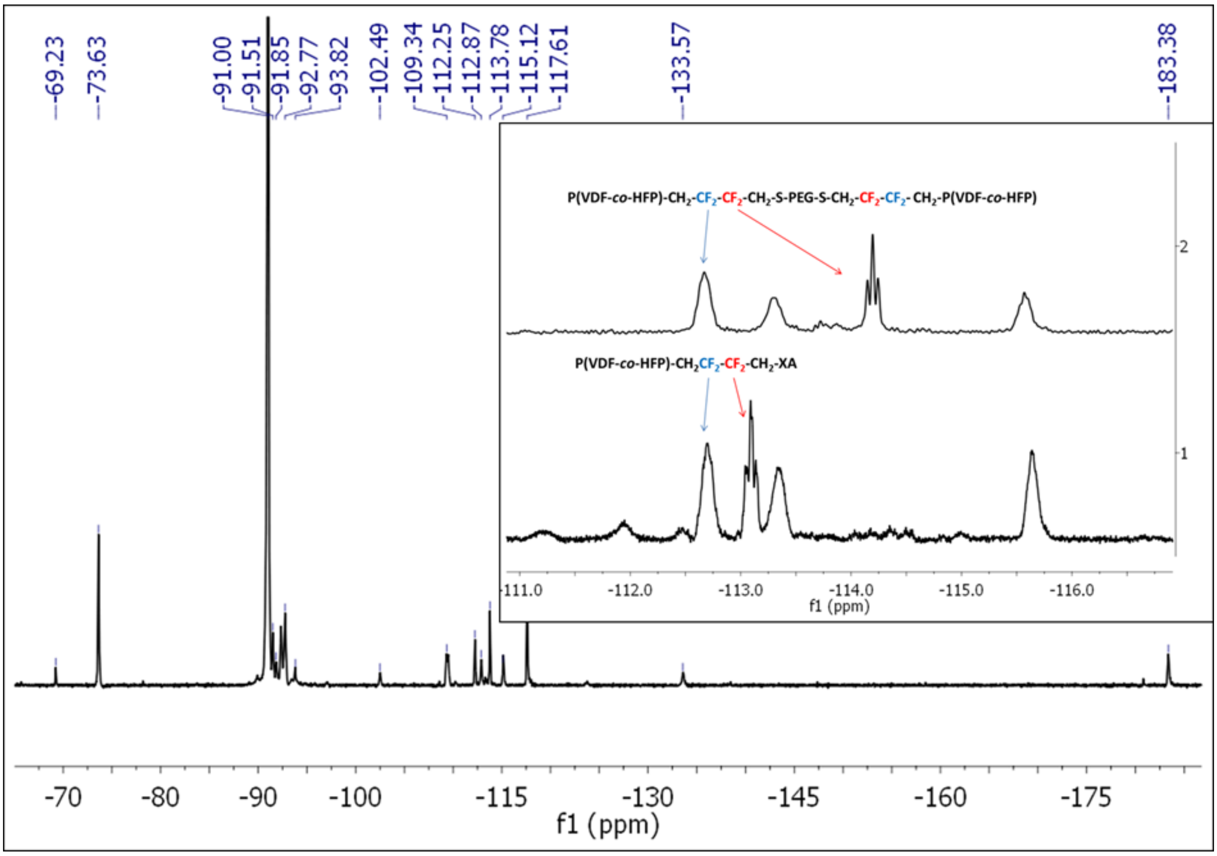

Figure S7. ^{19}F NMR spectrum ($(CD_3)_2SO$, 376 MHz) of P(VDF$_{51}$-*co*-HFP$_4$)-*b*-PEG$_{136}$-*b*- P(VDF$_{51}$-*co*-HFP$_4$). Inset: Shift of signals after the "one-pot" (aminolysis and thia-Michael) coupling reaction.

S8 Determination of –CH$_2$-CF$_2$H end group proportion from ^{1}H NMR.

$$(\%) - CH_2 - CF_2H = \frac{\int_{6.05}^{6.50} (-CH_2 - CF_2H + -CF_2 - CFH(CF_3) + -CF(CF_3)CF_2H)}{\frac{1}{3}\int_{1.71}^{1.87} -CF_2 - CH_3 + \int_{6.05}^{6.50} (-CH_2 - CF_2H + -CF_2 - CFH(CF_3) + -CF(CF_3)CF_2H) + \frac{1}{2}\int_{4.02}^{4.20} -CF_2 - CH_2 - XA}$$

*Data extracted from Figure S1.

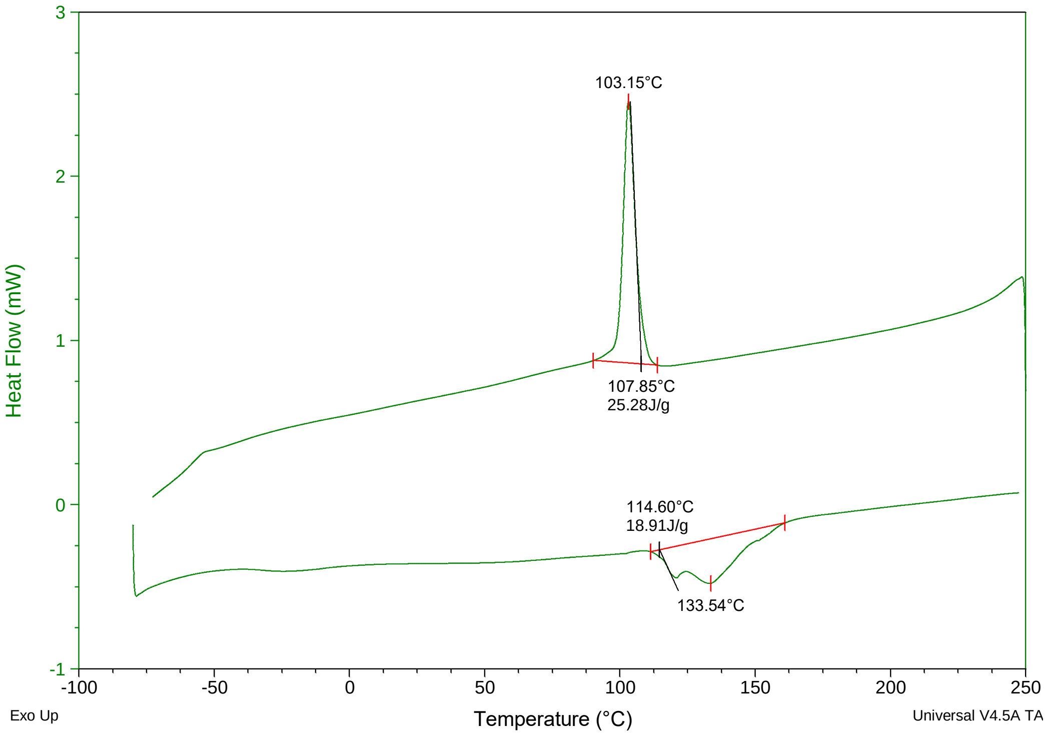

Figure S9. P(VDF-*co*-HFP) DSC thermograms. Second heating and cooling ramps.

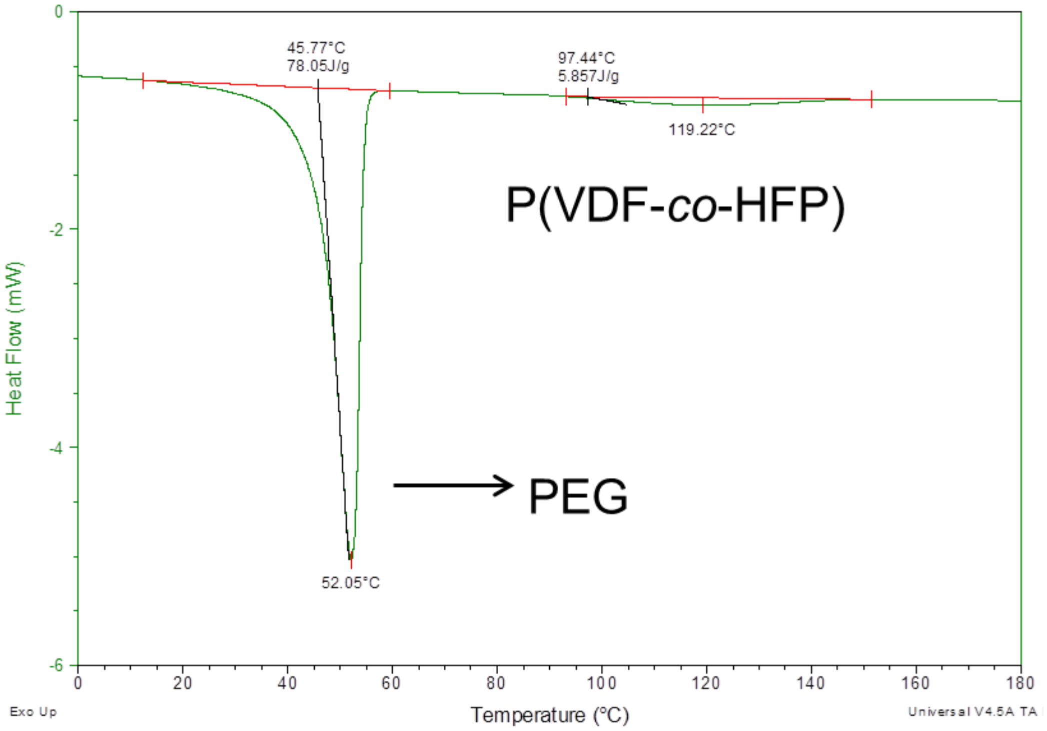

Figure S10. P(VDF-*co*-HFP)-*b*-PEG-*b*-P(VDF-*co*-HFP) DSC thermogram. Second heating ramp.

S11. Calculation of the degrees of crystallinity.

$$\chi_c(\%) = \frac{\Delta H_f}{\Delta H_{f^\circ}\ \phi_m} \times 100$$

Where ΔH_f is heat of melting (extracted from the DSC trace) and ΔH_{f° is a reference value and represents the heat of melting if the polymer were 100% crystalline (both in J/g). ϕ_m is the weight fraction of the different polymer forming the triblock copolymer.

ΔH_{f° of PVDF and PEG were extracted from the literature as 104.7 $J{\cdot}g^{-1}$ and 196.8 $J{\cdot}g^{-1}$ respectively.[1,2]

The molar mass of the triblock copolymer is estimated to be 14100 $g{\cdot}mol^{-1}$ and the Weight fraction of the PVDF and PEG blocks (ϕ_m) are 0.56 and 0.44 respectively.

χ_c PVDF = (5.857/(104.7·0.56))x100= 9.90%

χ_c PEG = (78.05/(196.8·0.44))x100=90.10%

1. Hietala, S.; Holmberg, S.; Karjalainen, M.; Na, J.; Paronen, M.; Serimaa, R. Structural investigation of radiation grafted and sulfonated poly (vinylidene fluoride), PVDF , membranes. *J. Mater. Chem.* **1997**, *7*, 721–726, doi:10.1039/A607675K.

2. Pielichowska, K.; Bieda, J.; Szatkowski, P. Polyurethane / graphite nano-platelet composites for thermal energy storage. *Renew. Energy* **2016**, *91*, 456–465, doi:10.1016/j.renene.2016.01.076.

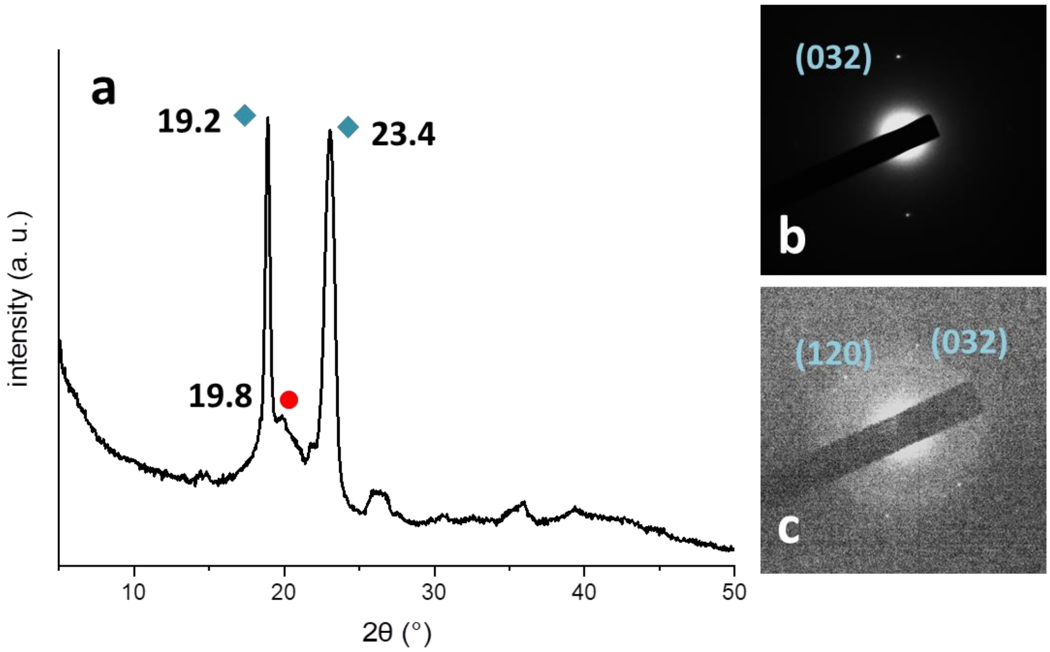

Figure S12. (a) XRD pattern of P(VDF-*co*-HFP)-*b*-PEG-*b*-P(VDF-*co*-HFP). Blue Rhombus and red dots correspond to PEG and PVDF characteristic diffraction signals respectively. (b, c) SAED patterns recorded during TEM analysis of ovoids and squares presented in figures 5a 6e and 6f respectively.

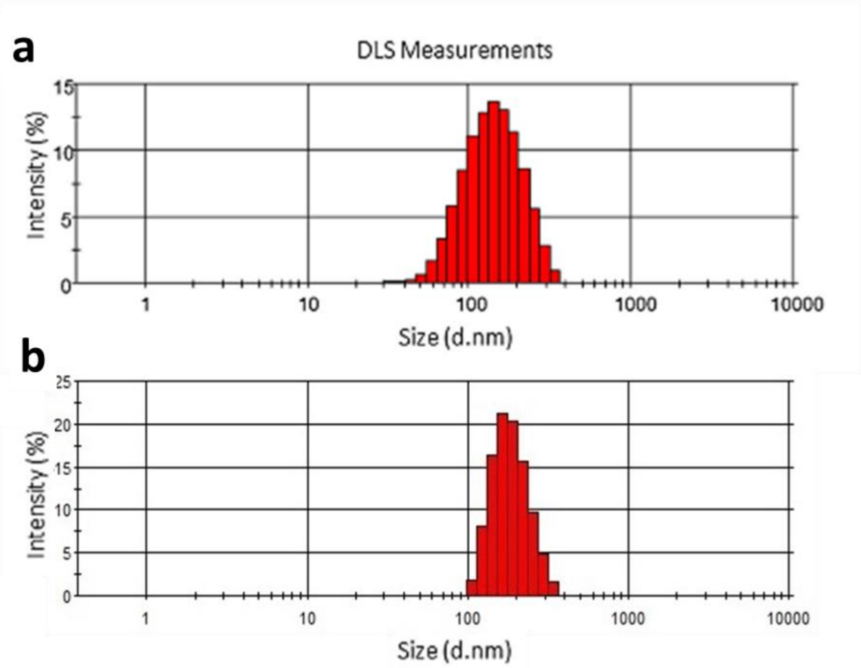

Figure S13. DLS characterization of P(VDF-*co*-HFP)-*b*-PEG-*b*-P(VDF-*co*-HFP) aggregates prepared by (a) Thin film hydration in water and (b) nanoprecipitation of a DMF solution in ethanol as non-solvent.

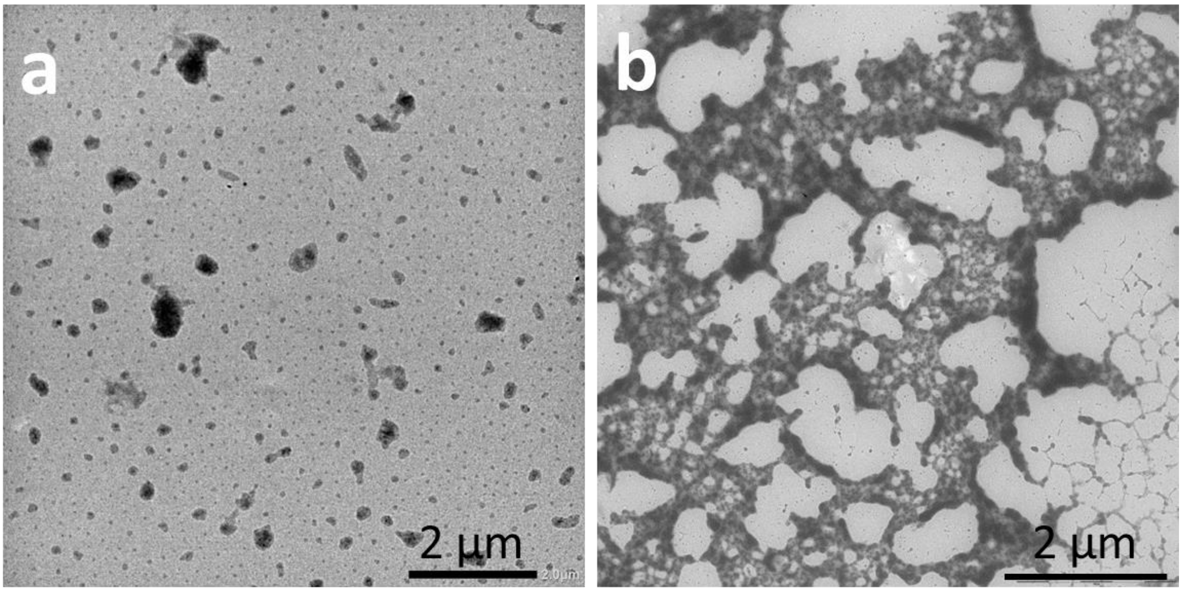

Figure S14. TEM images of self-assembled structures obtained by micellization of 1 mg mL^{-1} solutions of the P(VDF-*co*-HFP)-*b*-PEG-*b*-P(VDF-*co*-HFP) triblock copolymer in THF employing: (a) ethanol, (b) water, as selective solvent for PEG. Final concentration of all samples = 0.14 mg mL^{-1}, solvent: selective solvent final ratio=1:6.

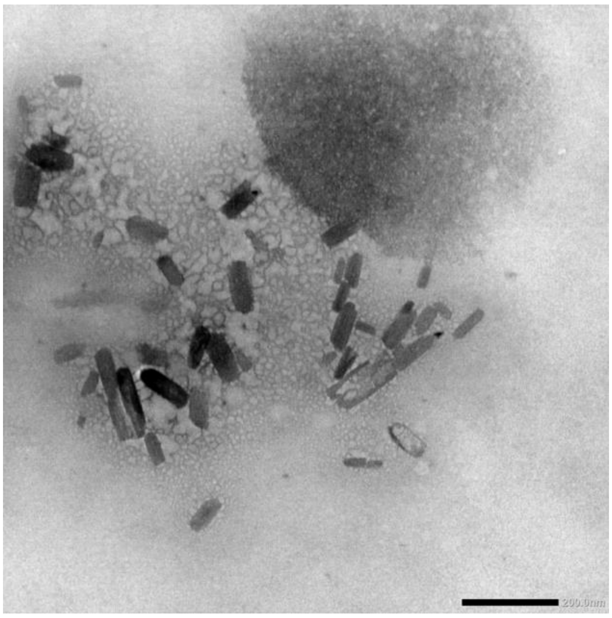

Figure S15. TEM image of the self-assembled structures obtained after micellization of a DMF solution using ethanol as non-solvent and thermal annealing treatment.
